# Editorial: Ubiquitin Code: From Cell Biology to Translational Medicine

**DOI:** 10.3389/fcell.2021.791967

**Published:** 2021-10-28

**Authors:** Cui Hua Liu, Yu Rao, Daming Gao, Lixin Wan, Lingqiang Zhang

**Affiliations:** ^1^CAS Key Laboratory of Pathogenic Microbiology and Immunology, Institute of Microbiology, Center for Biosafety Mega-Science, Chinese Academy of Sciences, Beijing, China; ^2^Savaid Medical School, University of Chinese Academy of Sciences, Beijing, China; ^3^Ministry of Education Key Laboratory of Protein Sciences, Ministry of Education Key Laboratory of Bioorganic Phosphorus Chemistry and Chemical Biology, School of Pharmaceutical Sciences, Tsinghua University, Beijing, China; ^4^State Key Laboratory of Cell Biology, Shanghai Institute of Biochemistry and Cell Biology, Center for Excellence in Molecular Cell Science, Chinese Academy of Sciences, Shanghai, China; ^5^Department of Molecular Oncology, H. Lee Moffitt Cancer Center and Research Institute, Tampa, FL, United States; ^6^State Key Laboratory of Proteomics, Beijing Proteome Research Center, National Center for Protein Sciences (Beijing), Beijing Institute of Lifeomics, Beijing, China

**Keywords:** ubiquitin code, ubiquitination, deubiquitination, disease, PROTAC technology

## Introduction

Ubiquitination is an important post-translational modification that involves the reversible conjugation of single ubiquitin (Ub) or various kinds of poly-ubiquitin chains (polyUb). Ubiquitination is carried out by the sequential actions of three enzymes including Ub-activating enzyme (E1), Ub-conjugating enzyme (E2) and Ub ligase (E3) to covalently link Ub to target protein. Ubiquitination can be classified as monoubiquitination, multi-monoubiquitination, and polyubiquitination according to the number and topology of ubiquitin molecules that are conjugated to the substrate. When polyUb chains are assembled, all seven lysine residues (K6, K11, K27, K29, K33, K48, and K63) and the N-terminal methionine residue on the proximal Ub are accessible by the distal Ub, allowing the assembly of eight homotypic and multiple-mixed conjugates. On top of these, the Ub moiety is often subjected to post-translational modifications. Hence, such three-layered construction of the ubiquitination modification is featured with great complexity and versatility, which is referred to as the ubiquitin code. Ubiquitination is carried out upon substrate proteins by E2/E3 ligase complexes (corresponding to “writers”) and removed from substrates by deubiquitinating enzymes (DUBs) (corresponding to “erasers”). The accurate assembly and interpretation of ubiquitin code is vital to protein homeostasis such as protein turnover, subcellular localization, interactions and activities. Therefore, ubiquitination is involved in all cellular processes and the deregulation of ubiquitination and deubiquitination is linked to the pathogenesis of a number of human diseases, such as cancer, neurodegenerative, infectious, inflammatory and metabolic disorders (Deng et al., 2020; Mulder et al., 2020).

Recently, the in-depth mechanistic studies of several key E3s or DUBs in conjunction with the emergence of high-throughput and novel technologies such as proteome microarray and PROteolysis-TArgeting Chimeras (PROTACs) have shed light on the underlying biochemical mechanisms as well as physiological and pathological functions of ubiquitination (Hu and Crews, 2021; Ramachandran and Ciulli, 2021). This Frontiers Research Topic comprises a series of reviews and original research articles highlighting the current understanding on the functions and mechanisms involved in protein de/ubiquitination and human diseases.

## Regulatory Roles of Ubiquitination in Physiological and Pathological Processes

The majority of the research articles in this Research Topic addresses the regulatory functions and mechanisms of a number of E3s and DUBs during physiological and pathological processes. The study by Gong et al. demonstrates that the CUL5-ASB6 E3 ligase complex that promotes p62/SQSTM1 ubiquitination and degradation to regulate cell proliferation and autophagy. Their study identified a new molecular mechanism regulating p62 stability, which may provide new insights into the delicate control of cell proliferation-autophagy in physiological and pathological settings. The study by Liao et al. aimed to investigate the regulatory effect and the underlying mechanisms of OTUB1, a deubiquitinating enzyme, on prostate cancer (PrCa) cell proliferation. They demonstrate that OTUB1 promotes the proliferation and progression of PrCa via deubiquitinating and stabilizing Cyclin E1. When blocking OTUB1/Cyclin E1 axis or applying the CDK1 inhibitor RO-3306, the occurrence and development of PrCa were significantly repressed. This finding indicates that OTUB1/Cyclin E1 axis might provide a new and potential therapeutic target for PrCa. In another study, Liu Y. et al. discovered that TNFAIP1, an adaptor protein of Cullin3 E3 ubiquitin ligases, coordinates with Cullin3 to mediate RhoB degradation through the ubiquitin proteasome system. They further show that Cullin3-TNFAIP1 E3 ligase controls inflammatory response in hepatocellular carcinoma cells via ubiquitination of RhoB. Their findings reveal a novel mechanism of RhoB degradation and provide a potential strategy for anti-inflammatory intervention of tumors by targeting TNFAIP1-RhoB axis. Another study by Guo et al. unveils that targeting the E3 ubiquitin ligase RFWD2 (also named COP1) could be an effective strategy to inhibit cellular proliferation and overcome drug resistance to proteasome inhibitor in multiple myeloma (MM).

Dysregulation of the ubiquitin-proteasome system itself could influence its function and be associated with multiple cellular homeostasis and disease progress signatures. In a study by Zhou, Yu et al. the authors addressed the question whether the stability and its biological function of Cereblon (CRBN), a substrate receptor of cullin 4-RING E3 ligase (CRL4), could be modulated by caspases. They found that Caspase-8 inhibition prevents the cleavage and degradation of CRBN and potentiates its biological function, suggesting that administration of Caspase-8 inhibitors might enhance the overall effectiveness of Len-based combination therapy in myeloma. With an aim to explore the ubiquitin modification features of clear cell renal cell carcinoma (ccRCC) and to elucidate the role of such ubiquitin modifications in shaping anti-tumor immunity and individual benefits from immune checkpoint blockade (ICB), Zhou, Lu et al. conducted RNA-seq analysis to elucidate the potential link between ubiquitin modification and immune infiltration landscape of ccRCC. Their study provides a new assessment protocol for the precise selection of treatment strategies for patients with advanced ccRCC through constructing a ubiquitin score to evaluate individual patients' ubiquitination outcome. Similarly, another original research by Wu et al. also addresses the molecular characteristics and prognostic value of ubiquitin in ccRCC, and they developed an individualized ubiquitin prognostic signature for ccRCC and confirmed that the signature is an independent prognostic factor related to the prognosis of ccRCC patients, which may help to reveal the molecular mechanism of ccRCC and provide potential diagnostic and prognostic markers for ccRCC. In the study by Li et al. low-dose DNA demethylating agent decitabine was found to enhance the expression of β-TrCP, a substrate recruiting subunit of the Skp1, Cullin 1, F-box-containing complex (SCF complex). Elevated β-TrCP in turn promotes the proteolysis of IκBα and subsequent NF-κB activation in IFN-γ^+^ CD4^+^ T cells, which improves anti-tumor immunity.

Except for the above original research papers, there are also a few review articles that summarize recent progress in the regulatory function of several important E3s or their components. For example, Wang L. et al. reviewed the function and molecular mechanisms of Deltex family ubiquitin E3 ligases in development and disease, providing insights into future research directions and potential strategies in disease diagnosis and therapy. Another review by Bodrug et al. summarized the intricate regulatory mechanisms of the Anaphase-Promoting Complex/Cyclosome (APC/C) and its role in chromatin regulation. In a comprehensive review, Sun et al. summarize the molecular characteristics of FBXW7, an F-box protein serving as the substrate recognition component of SCF E3 ubiquitin ligase. They also provided future perspectives to further elucidate the role of FBXW7 in the regulation of a variety of biological processes and tumorigenesis, and to design a number of approaches for FBXW7 reactivation in a subset of human cancers for effective anticancer therapy.

Instead of focusing on the regulatory mechanisms of individual ubiquitination-regulating enzymes, there are several review articles addressing the regulation of cellular functions by the ubiquitiantion process as a whole. For example, in a review article, Lei et al. provided an in-depth understanding of the molecular mechanisms by which ubiquitination regulates small GTPases, thus revealing novel insights into the membrane trafficking process. In another review, Wang X. et al. summarized the current findings of ferroptosis surrounding the viewpoint of ubiquitination regulation, highlighting the potential effect of ubiquitination modulation on the perspective of ferroptosis-targeted cancer therapy.

Although this collection centers on ubiquitination, neddylation, a ubiquitin-like modification that earmarks substrate proteins with the small ubiquitin-like protein NEDD8, is also part of this Research Topic due to its roles in controlling the Cullin-RING and Smurf1 ubiquitin E3 ligases. Jiang et al. updated our current understand of neddylation in tumor-associated macrophages, and Gai et al. summarized the approaches developed to target the neddylation pathway. The study by Du et al. reported *PTEN* deficiency as a key mechanism that contributes to the neddylation inhibitor MLN4924 resistance in breast cancer cells.

## Application of Protein Microarray in Deciphering Ubiquitin Code

Linear ubiquitin chain assembly complex (LUBAC) catalyzes linear ubiquitination, while the deubiquitinase OTULIN exclusively cleaves the linear ubiquitin chains (Oikawa et al., 2020). To expand understanding of the substrates and pathways of linear ubiquitination, Zhou, Ge et al. used a human proteome microarray (a high-throughput technology that allows systematically screening up to 20,000 proteins) to conduct global screening of LUBAC- and OTULIN- interacting proteins. They identified many potential new interacting proteins of LUBAC and OTULIN, which may function as novel regulators or substrates of linear ubiquitination. Their results suggest that linear ubiquitination may have broad cellular functions and is associated with diverse signaling pathways, and provide accessible data for the interacting proteins of LUBAC and OTULIN, which helps guide further studies to broaden our understanding on linear ubiquitination.

## Degradation of Target Proteins and Related Drug Research by PROTAC Technology

PROTACs is an emerging and promising approach to target intracellular proteins for ubiquitination-mediated degradation, including previously undruggable protein targets, such as transcriptional factors and scaffold proteins. To date, plenty of PROTACs have been developed to degrade various disease-relevant proteins, such as estrogen receptor (ER), androgen receptor (AR), BTK, RTK, and CDKs, etc. Notably, ER and AR targeting PORTAC molecules have entered phase II clinical studies. More recently, the third generation light-controllable PROTACs have been developed to overcome the limitation of the on-target off-tissue and off-target effect of this technology (Hu and Crews, 2021; Ramachandran and Ciulli, 2021). A review by Liu J. et al. summarized the emerging light-controllable PROTACs and the prospective for other potential ways to achieve temporospatial control of PROTACs.

## Conclusions

The collection of articles in this Research Topic provides a number of key findings on the regulatory functions and mechanisms of ubiquitination system in recent years, presenting compelling evidence for a critical role of ubiquitination in cell biology and human diseases and suggesting that targeting the ubiquitination machinery could be an effective strategy for treating certain diseases. It should be pointed out that the coverage is far from complete in this Research Topic, and there are some other equally important questions that are not covered in this issue but warrant future in-depth investigation. For example, how the ubiquitin code is dynamically edited and precisely interpreted in different cellular microenvironment? What are the writers, readers and erasers of each type of the polyUb chains as well as the branched ubiquitin chains in cells? What are the new modifications, linkages and targets of ubiquitin molecule? What are the novel host-regulating functions and unique biochemical mechanisms of bacterial/viral ubiquitin ligases and deubiquitinases? Hence, it is clear that ubiquitination remains a dynamic field, and we will see many more exciting discoveries of how ubiquitianation is assembled and dis-assembled to dynamically fine-tune normal cellular functions and thus affects multiple disease progress in the near future.

## Author Contributions

CL and LZ wrote the original manuscript. All authors reviewed, edited the manuscript, and approved the submitted version.

## Funding

CL was supported by National Key Research and Development Program of China (2017YFA0505900) and National Natural Science Foundation of China (Grant Nos. 81825014 and 31830003).

## Conflict of Interest

The authors declare that the research was conducted in the absence of any commercial or financial relationships that could be construed as a potential conflict of interest.

## Publisher's Note

All claims expressed in this article are solely those of the authors and do not necessarily represent those of their affiliated organizations, or those of the publisher, the editors and the reviewers. Any product that may be evaluated in this article, or claim that may be made by its manufacturer, is not guaranteed or endorsed by the publisher.

## References

[B1] Deng L. Meng T. Chen L. Wei W. Wang P. (2020). The role of ubiquitination in tumorigenesis and targeted drug discovery. Signal Transduct. Target Ther. 5:11. 10.1038/s41392-020-0107-0 32296023 PMC7048745

[B2] Hu Z. Crews C. M. (2021). Recent developments in PROTAC-mediated protein degradation: from bench to clinic. Chembiochem. 22, 1–23. 10.1002/cbic.202100270 34494353 PMC9395155

[B3] Mulder M. P. C. Witting K. F. Ovaa H. (2020). Cracking the ubiquitin code: the ubiquitin toolbox. Curr. Issues Mol. Biol. 37, 1–20. 10.21775/cimb.037.001 31674341

[B4] Oikawa D. Sato Y. Ito H. Tokunaga F. (2020). Linear ubiquitin code: its writer, erasers, decoders, inhibitors, and implications in disorders. Int. J. Mol. Sci. 21:3381. 10.3390/ijms21093381 32403254 PMC7246992

[B5] Ramachandran S. Ciulli A. (2021). Building ubiquitination machineries: E3 ligase multi-subunit assembly and substrate targeting by PROTACs and molecular glues. Curr. Opin. Struct. Biol. 67, 110–119. 10.1016/j.sbi.2020.10.009 33271439

